# Stand-Sit Microchip for High-Throughput, Multiplexed Analysis of Single Cancer Cells

**DOI:** 10.1038/srep32505

**Published:** 2016-09-01

**Authors:** Lisa Ramirez, Jason I. Herschkowitz, Jun Wang

**Affiliations:** 1 Multiplex Biotechnology Laboratory, Department of Chemistry, University at Albany, State University of New York, Albany, NY 12222, USA; 2 Cancer Research Center, University at Albany, State University of New York, Rensselaer, NY 12144, USA; 3 Department of Biomedical Sciences, University at Albany, State University of New York, Albany, NY 12222, USA.

## Abstract

Cellular heterogeneity in function and response to therapeutics has been a major challenge in cancer treatment. The complex nature of tumor systems calls for the development of advanced multiplexed single-cell tools that can address the heterogeneity issue. However, to date such tools are only available in a laboratory setting and don’t have the portability to meet the needs in point-of-care cancer diagnostics. Towards that application, we have developed a portable single-cell system that is comprised of a microchip and an adjustable clamp, so on-chip operation only needs pipetting and adjusting of clamping force. Up to 10 proteins can be quantitated from each cell with hundreds of single-cell assays performed in parallel from one chip operation. We validated the technology and analyzed the oncogenic signatures of cancer stem cells by quantitating both aldehyde dehydrogenase (ALDH) activities and 5 signaling proteins in single MDA-MB-231 breast cancer cells. The technology has also been used to investigate the PI3K pathway activities of brain cancer cells expressing mutant epidermal growth factor receptor (EGFR) after drug intervention targeting EGFR signaling. Our portable single-cell system will potentially have broad application in the preclinical and clinical settings for cancer diagnosis in the future.

A tumor is a highly heterogeneous society that often consists of several cell subtypes varying in genome, phenotype, and function^1^. Subpopulations of tumor cells can harbor different tumorigenic potential, and may be generated by continuous genetic and epigenetic changes as well as interactions within the tumor microenvironment. All together, these maintain hierarchical organization in a tumor and promote tumor progression. Such intratumoral heterogeneity poses a major challenge to cancer diagnosis and treatment, since differential regulation of signaling networks within the tumor may underlie the inability of current therapies to achieve long-term remissions^2^,^3^. Understanding the molecular signatures and phenotypic properties of tumor subpopulations would be of great value in improving diagnosis, accelerating drug discovery, and overcoming treatment resistance.

Progress in characterizing heterogeneous tumor samples has been largely propelled by the advancement of high-throughput, multiplexed platforms for single-cell analysis^4^. In recent years, some emerging single-cell tools have been used to investigate the entire genome and transcriptome of single cells with statistically large samples of cells^5^,^6^. Heterogeneity in cell signaling represented by functional proteins is particularly notable since many cancer drugs are developed to target oncogenic signaling but fail to meet expectations. Functional proteins including signaling kinases, surface receptors and secreted proteins are useful indicators of a cell’s physiological state. In many cases they reflect the cell’s immediate response to its environment, and are also directly involved in carrying out cellular functions such as adhesion, migration, etc. It is known that cancer cells may exhibit disparate regulation of oncogenic pathways and surface marker expression, and multiplexed single cell proteomic assays allow for the investigation of these aspects simultaneously, thus they possess a significant advantage over singleplexed counterparts used in studying cancer cell signaling^7^,^8^,^9^. Multiplexed screening assays have also been developed for profiling large collections of potential drug targets^10^,^11^. In addition, high-throughput multiplexed single-cell assays enable the study of protein-protein correlations and mapping of the population-wide change of cell characteristics^12^. Quantification of protein fluctuations at the single-cell level has also been used to resolve the structure of signaling networks^7^.

Unfortunately, little effort has been done to take heterogeneity into consideration in the clinical treatment of cancer, mainly due to the lack of appropriate multiplexed single-cell tools that operate in a field setting. Currently available multiplexed single-cell tools fall under microfluidic platforms and cytometry tools including flow cytometry and time-of-flight mass cytometry (CyTOF). Fluorescence-based flow cytometry has been implemented as the major cell biology instrument for decades and is capable of routinely analyzing 3 or more markers^13^. The multiplexity has been significantly enhanced by CyTOF, which measures over 40 proteins in single cells using isotope mass labeling^11^. Such technologies are not portable and operable in a field setting. Microfluidics brings enormous opportunities to point-of-care diagnosis by minimizing the analytical platforms while retaining capabilities of the conventional counterparts. The microengraving technique utilizes a microchip with many nano-wells enclosed by an antibody-coated coverslip for detecting secreted proteins^14^,^15^. This platform can also analyze the secretion kinetics of T cells, with the option of recovering the assayed cells. Another important tool is single-cell western blotting which is more useful for detection of intracellular proteins, although the sensitivity has not been comparable to flow cytometry yet^16^. The single-cell barcode chip encompasses the ability to measure both secreted proteins and intracellular phosphoproteins with a multiplexity up to 45^17^,^18^. It has been applied in studies of macrophage secretion, T cell immunotherapy, cancer cell signaling and cell-cell communications^19^,^20^,^21^,^22^,^23^. This technique integrates a high-density antibody array into a microchip and usually uses pneumatic valves to manipulate single cells and on-chip assay steps, and requires external facilities to support pressurization. The new versions of the barcode microchips have simplified the chip design, so the operation is not dependent on microfluidic valves^17^,^18^.

Herein we introduce a portable microfluidic system that leverages the merits of the single-cell barcode chip and is designed towards point-of-detection applications. A significant improvement from the prior design has been made, so that the on-chip single-cell assay is more resilient and robust to various tasks. The most distinctive characteristic of the chip is having an adjustable inner volume that may be expanded or compressed by application of mechanical force. Another notable innovation is that the typical microfluidic features such as microchannels are only formed under certain threshold of external force. The microchip operation permits a switch from a bulk assay at the “stand” state to single-cell assay at the “sit” state. By varying forces on the chip, the typical controls carried out by large-scale microfluidic valve circuits can be also achieved on our “stand-sit chip” (SSC). With these designs, the operation of the SSC platform only requires simple instruction. We have fully validated the SSC technology for assaying multiple cancer-relevant proteins, and demonstrate its applications in studying oncogenic signaling of cancer stem cells as well as drug treated cancer cells. With the valve-less SSC, we are able to profile both secreted proteins and intracellular proteins from the same single cells on various sample sources. The SSC is a portable, reliable single-cell tool that may advance high-throughput, multiplexed single-cell analysis from laboratory-based technology to point-of-care diagnostics.

## Results

### Design and fabrication of the stand-sit microchip

The concept of the stand-sit chip (SSC) design is to have a stand-alone, portable microchip system towards point-of-care diagnosis that permits high-throughput, multiplexed detection of single cell proteins in a field setting. The SSC is comprised of two parts: a PDMS replica and an antibody barcode slide. Different from most other microchips, the volume enclosed by the PDMS replica and the slide is adjustable subject to the external mechanical force on the PDMS. The typical single-cell assay processes including cell culture, exchange of medium, cell lysis, and multiple steps of sandwich enzyme-linked immunosorbent assay (ELISA) are all integrated into the system without requiring microfluidic valves. We take advantage of the elasticity of PDMS to control fluidic flow and exchange of materials with the outside environment, thus obviating the need for additional equipment, such as external pneumatic pumps, normally connected with a single-cell microchip.

**Figure 1:**
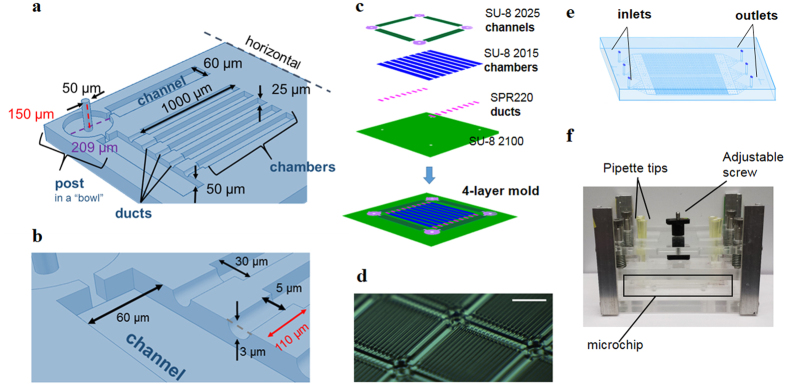
Design of portable single-cell microchip system. (**a,b**) Features on the PDMS replica with corresponding dimensions. (**c**) Scheme for the fabrication of a 4-layer microchip mold using SU-8 2015, 2025, 2100, and SPR220 photoresists. Four layers of photoresists are stacked on one another and carefully aligned during the fabrication process on a 4″ silicon wafer. The bottom layer at a thickness of 100 μm is used as the base for all the other layers, which is translated to be the PDMS horizontal. Once it is developed following standard photolithography protocol, the second layer for ducts made of SPR 220 photoresist is fabricated on the previous layer. Similar procedures are followed for adding the third and the fourth layers corresponding to cell chambers and channels, respectively. While the features on all four layers are distinctive, only the posts on the photomasks are hollowed out and aligned throughout all layers, resulting in long PDMS posts with a height of 150 μm after casting. (**d**) Microchip mold on a silicon wafer showing cell chamber arrays connected to microchannels in a grid design (scale bar = 500 μm). (**e**) Diagram of the whole stand-sit chip (SSC). All the features on PDMS are fabricated together from one mold, which avoids the downstream multistep alignment for constructing multilayer PDMS devices in most single cell microchips and simplifies the microchip fabrication process as well as operation process. (**f** ) Mechanical clamp system for the operation of the SSC showing the adjustment screw to vary the external forces to switch between chip states, and pipette tips connected to the inlets and outlets.

The key elements that enable large-scale manipulation of single cells are posts and ducts, with the latter connected to microchannels and cell chambers (Fig. 1). These 4 features are on the same PDMS replica face; the posts project out of the PDMS horizontal while the others are engraved on the horizontal. The tall and thin posts are PDMS bas-relief structures serving as elastic pillars that support the body of the chip and adjust the space (up to 100 μl) between the PDMS horizontal and the glass slide. We intentionally design a “bowl” shape to embed the bent posts under stress (Fig. 1a), so that the posts after deformation would not create a local lump that would cause unevenness at the corners of the cell chamber units and cause leakage. Only when the tall posts entirely collapse under a certain amount of force can the other PDMS features be enclosed with the glass slide to form ducts, microchannels and cell chambers. Thus, the posts make it possible to switch between single-cell manipulation and bulk assay processes (the advantages will be detailed later). The ducts are shallow and short conduits connecting cell chambers to microchannels (Fig. 1b), and the height is varied with the exerted force. The ducts are used to control content exchange between the cell chambers and microchannels. The microchannels form a grid throughout the whole chip (Fig. 1d) are directly connected to the inlets and outlets. Each grid contains 16 cell chambers, and the whole SSC is comprised of 377 grids, or about 6000 cell chambers for high-throughput single cell analysis.

Three states of operation including “stand”, “sit-open” and “sit-closed” are defined according to the extent of PDMS deformation, or the strength of external force applied to the PDMS while the microchip is held together by a mechanical clamp (Fig. 1f). Switching between states is done simply by adjusting the screws of the clamp and hence the force exerted on the PDMS. In each state, the material exchange processes are varied for specific purposes and stages of the single-cell assay procedure (Table S1). Material exchange in the SSC can occur via two mechanisms. The first is diffusion through the conduits which is dependent on the molecular weight. The second is fluidic convection initiated by transient change of external force on the PDMS, because the microchannels and chambers are interconnected and their volumes are changed with the external force.

**Figure 2:**
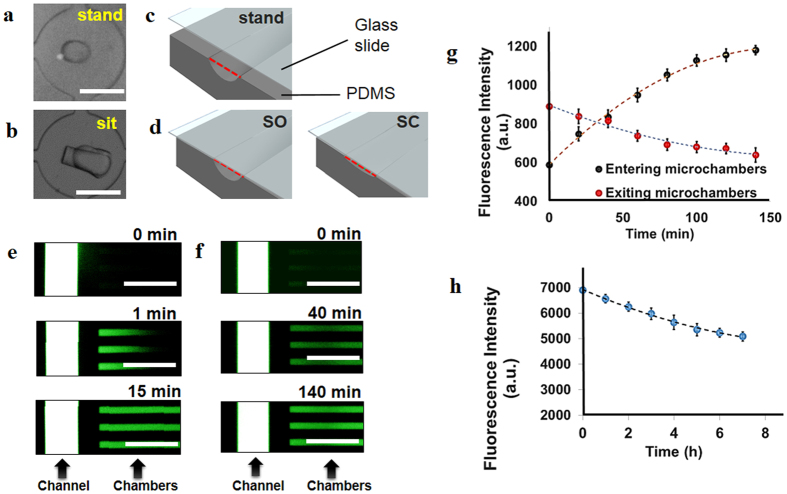
(**a,b**) Characterization of material exchange processes related to stand-sit states of the microchip. Top-view images of a standing post (**a**) and sitting post (**b**) under a bright field microscope. (**c**) At the stand state, ducts are not formed because the barcoded glass slide is not touching the PDMS horizontal. (**d**) At the sit state, ducts may be open (sit-open, SO) or closed (sit-closed, SC). (**e**) Convective mass transfer of Dylight 488 from channels to chambers induced by the quick transition from SC to SO, which pushes dye molecules into chambers. (**g**) Diffusion of Dylight 488 quantitated by fluorescence intensity with time while the chip is in the SO state. The plot shows two cases for diffusion: net entry of dye molecules into chambers when dye concentration is higher in channels, and net exit of molecules into channels when dye concentration is higher in chambers. (**h**) Fluorescence intensity of SA-647 in microchambers diminishes over time while the chip is in the SO state, and dye concentration within microchambers is higher than that of channels. All error bars denote standard deviation from the mean. All scale bars are 100 μm.

At the “stand” state, no force is exerted by the clamp, and the posts remain upright (Fig. 2a). At this point, the distance between the barcoded slide and the PDMS horizontal is 100 μm, which is large enough to control bulk flow of cell suspensions and other reagents for sandwich ELISA from the inlets to the outlets of the SSC simply by pipetting. At the stand state, there are no microchannels or cell chambers formed, thus the chip is suitable for bulk assays similar to those conducted in well plates.

With a mild force, the chip assumes the “sit-open” state where the ducts are formed and kept open. The microchannels and cell chambers are enclosed, and portions of fluidic contents such as single cells are encapsulated in the cell chambers. At this state, the exchange of contents in microchannels is directed by gravity-induced flow, while the material exchange between cell chambers and microchannels is achieved by diffusion. We first characterized the rate of material exchange in microchannels using food dye (Fig. S1). We found that 8 min was enough to completely exchange the solutions in the microchannels by the fluidic flow initiated by gravity when the whole clamp system was tilted. Thus, whenever we introduced new solutions into the chip, we used a minimum of 10 min for complete exchange of contents within the microchannels of the chip. In addition, we tested the performance of the ducts by characterizing the diffusion time of small molecules between cell chambers and microchannels under static conditions. We used Dylight 488 (MW = 1 kDa) to visualize the diffusion for the following cases: (a) when solute concentration is higher in microchambers than in channels, and (b) when the channels have a higher concentration of solute (Fig. 2f). We found that the concentration of Dylight 488 in chambers falls to 50% in ~60 min for the former case while the concentration of Dylight increases by 50% in ~50 min for the latter case (Fig. 2g). After 2 h, diffusion has reached 90% completion. We also characterized the diffusion of larger molecules, represented by Streptavidin-Alexa 647 (SA-Alexa 647; MW = 60 kDa), at the “sit-open” state (Fig. 2h). Streptavidin concentration in the chambers decreases by ~10% after 4 h of diffusing through ducts from chambers into microchannels. These results indicate that only a negligible portion of proteins from single cells might be lost during the on-chip assay.

With a high force applied by turning the adjustable screw of the clamp, the microchip is switched to the “sit-closed” state where the ducts may be narrowed or completely obscured (Fig. 2d). At this state, the chambers are completely inaccessible, while the fluidic flow by gravity in microchannels is not significantly influenced. Cells can be incubated in the chambers for hours without the influence of the fluidic flow in the microchannel environment, and the secreted proteins are captured by the antibody arrays aligned with every chamber. The quick switching between “sit-open” and “sit-closed” states leads to fast content exchange between chambers and microchannels within one minute (Fig. 2e). This operation is adopted to lyse cells by drawing lysis buffer from microchannels in single-cell assays.

**Figure 3:**
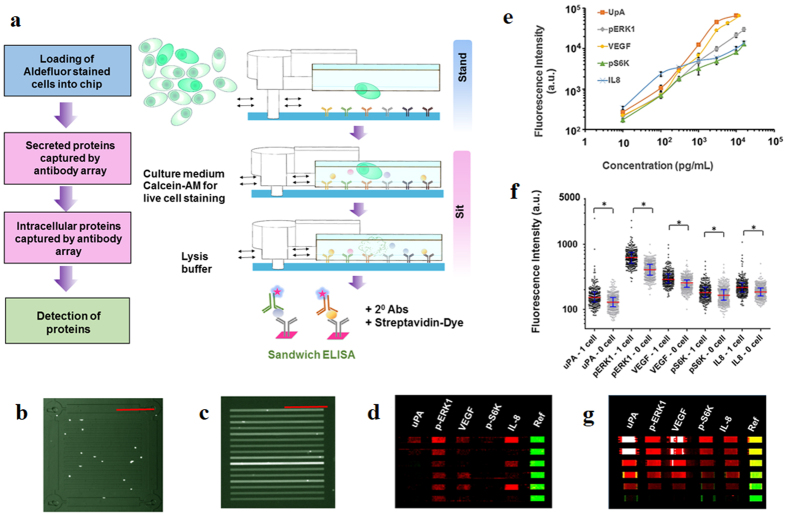
Integrated single-cell Aldefluor assay and functional protein assay. (**a**) Scheme of the experimental procedure. Aldefluor-stained cells are loaded while the chip is in the stand state. Conversion to the sit state is used to isolate single cells and perform other on-chip steps of the assay. Sandwich immunoassay is completed by adding biotinylated secondary antibodies and fluorophore-labeled streptavidin. (**b**,**c**) Cells are randomly distributed in microchambers before lysis (**b**) and after lysis (**c**) by diffusion of concentrated lysis buffer from channels to chambers. (**d**) Examples of detected proteins in red (Cy5 channel) and the reference signal in green (Cy3 channel) for single cells in each row. (**e**) Calibration curve for immunoassays performed on-chip using recombinant uPA, p-ERK1, p-S6K, VEGF, and IL-8. Error bars denote standard deviation from the mean. (**f** ) Fluorescence readouts obtained for different concentrations of recombinant protein used in calibration. (**g**) Scatter dot plot showing fluorescence data for secreted (uPA, VEGF, IL-8) and intracellular phosphorylated proteins (p-ERK1, p-S6K) from 1-cell experiments in the microchip compared to background levels from 0-cell experiments with *P* < 0.001 (***). All data are from the same chip to keep consistency. *P* values less than 0.05 (*) were considered statistically significant. All scale bars are 500 μm.

A multiplexed antibody array is integrated into each cell chamber to quantitate up to 10 proteins including intracellular proteins, surface markers and secreted proteins. This type of antibody microarray has been comprehensively characterized in our previous studies and surpasses commercial counterparts in that ours is >10 times smaller and is therefore compatible with single-cell protein assays^24^. Each element of the array detects one type of protein, and all the elements are spatially distributed with a reference in green fluorescence (Fig. 3). The measurement error by this antibody array is typically <10%^22^.

### Single-cell analysis of cancer stem cells

We demonstrate the application of the SSC in studying cancer stem cells. ALDH activity measured by the Aldefluor assay is widely considered as a marker for cancer stem cells (CSCs)^25^. Previous studies have shown that increased ALDH activity in normal and malignant human mammary cells is related to stem cell/progenitor properties^26^. MDA-MB-231 cells are used in our study because this cell line contains >90% CD44^+^ CD24^−^ cells^27^, and a subpopulation with the ALDH^high^ CD44^+^ CD24^−^ phenotype was associated with “stem-like” properties, such as increased invasion and migration *in vitro*, as well as increased tumorigenicity and metastasis *in vivo*^27^,^28^,^29^.

Typically, studies on CSCs are performed by sorting cells based on markers^30^ for stemness such as CD44, CD24, CD133, and ALDH by fluorescence activated cell sorting (FACS), followed by functional assays including Western blots, migration assays, etc. We sought to simplify the process of identifying and profiling prospective CSCs by integrating the Aldefluor assay with a multiplexed single-cell immunoassay into one platform. We demonstrate the capability of the SSC for co-detection of functional proteins uPA, VEGF, IL-8, p-ERK1, and p-S6K with ALDH activity in single cells. The relationship between ALDH markers and such signaling proteins has been investigated by comparing microarray protein detection readouts with the Aldefluor staining for all single cells.

The procedure for the on-chip integrated assay is shown in Fig. 3a. The assay is performed by first staining cells for ALDH activity, followed by loading the stained cells into the SSC. ALDH activity is profiled for every cell by imaging the whole chip upon loading cells into microchambers. Single cells are incubated while the chip is at the “sit-open” state so that during the incubation period, nutrition from medium in the microchannels diffuse into the chambers, and the unwanted staining reagents in chambers are cleaned out by either diffusion or convection. Switching between states also permits subsequent cell staining by live cell dye Calcein AM. The serial staining provides additional physiological parameters that many conventional assays lack. For instance, the calcein-AM staining allows us to assess the viability of cells at different stages of the assay. After 4 h incubation, fluorescence imaging of the chip allowed us to observe calcein AM-stained cells, so that we would only include viable cells for analysis of ALDH activity and functional protein profiling. This was followed by lysis of cells to release intracellular proteins, after which standard sandwich ELISA steps were performed for detection of captured proteins. All the detection steps after cell lysis can be done at the “stand” state where the operation is switched from single-cell assay to bulk assay.

We validated our technology first by detecting recombinant proteins at various quantities. We assayed three secreted proteins including urokinase-type plasminogen activator (uPA), vascular endothelial growth factor (VEGF) and interleukin 8 (IL-8), and two phosphorylated kinases (phospho(p)-ERK1 and p-S6K). The phosphorylated kinases are associated with MEK/ERK signaling, and the secreted proteins are generally indicative of invasive or aggressive cancer cells^31^,^32^,^33^,^34^. Cross-reactivity test shows the detection of individual proteins is orthogonal to each other (Fig. S3). Calibration curves demonstrate that the sensitivity of the antibody array is comparable with that using conventional ELISA assays based on well plates (Fig. 3e,f). We further compare the fluorescence intensities from one-cell chambers with those from zero-cell chambers. For all 5 proteins detected, the single cell signals are statistically higher than blanks (Fig. 3g).

**Figure 4:**
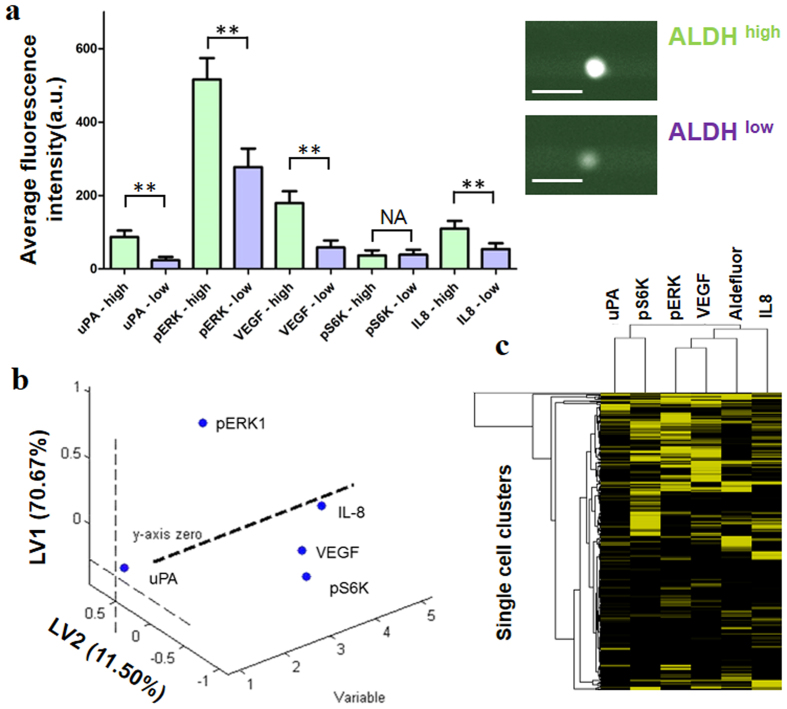
Relating ALDH activity to proteins in single cells. (**a**) Comparison of single-cell protein data from ALDH^high^ and ALDH^low^ populations of MDA-MB-231 (scale bar = 50 μm). The single-cell data for each protein are processed by setting a threshold at mean + 2 s.d. of 0 cell data. Any data lower than the threshold are considered as noise and the values are reassigned as 0. *P* values: *P* < 0.05 (*), *P* < 0.01 (**), with *P* *<* 0.05 considered significant. NS indicates no significant statistical difference. (**b**) Plot of latent variables 1 (LV1) and LV2 from a model built by partial least squares discriminant analysis (PLS-DA). The values projected on each LV are their percentile weight. (**c**) Heat map comparing relative levels of uPA, p-S6K, VEGF, p-ERK1, IL-8, and ALDH (Aldefluor) from single cells. Single cells and proteins/ALDH activity are clustered by Euclidean distance. Experiments are repeated two times, but the data in this figure are from the same chip to lower experimental variations.

ALDH subpopulations are identified by the differences of their fluorescence intensities. This imaging-based screening approach is conveniently modified from the conventional Aldefluor assay by FACS^35^,^36^. We took the top 20% high ALDH intensity as the ALDH^high^ subpopulation, and compared that with 20% subpopulation with the lowest ALDH activity, and this mirrors the sorting criterion previously used for Aldefluor-based FACS analysis on MDA-MB-231 cells^27^,^37^. We found that the protein levels of uPA, p-ERK and VEGF are significantly higher in the ALDH^high^ subpopulation (Fig. 4a). We sought for the best protein predictor for ALDH intensity using partial least square discriminant analysis (PLS-DA). PLS is a statistical prediction tool for dimension reduction of microarray data^38^. The PLS variables are constructed to maximize the sample covariance between the response (ALDH intensity) and the linear combination of the predictors (protein values)^39^. We found p-ERK1 weighs the highest to the latent variable 1 among the proteins we assayed, which implies that p-ERK1 signaling plays a key role in cancer stem cell activity (Fig. 4b). This is consistent with previous reports that maintenance and enhancement of the CSC state is correlated with activation of PI3K/Akt/mTOR pathway that involves ERK signaling^40^,^41^,^42^. Clustering of proteins and ALDH according to their Euclidean distances also shows the similar conclusion that the ALDH^high^ subpopulation is also active in PI3K pathway (Fig. 4c). Those cells also produce more VEGF, which means they are highly aggressive in promoting angiogenesis and meanwhile reproducing themselves.

### Single-cell analysis of drug treated cancer cells

**Figure 5:**
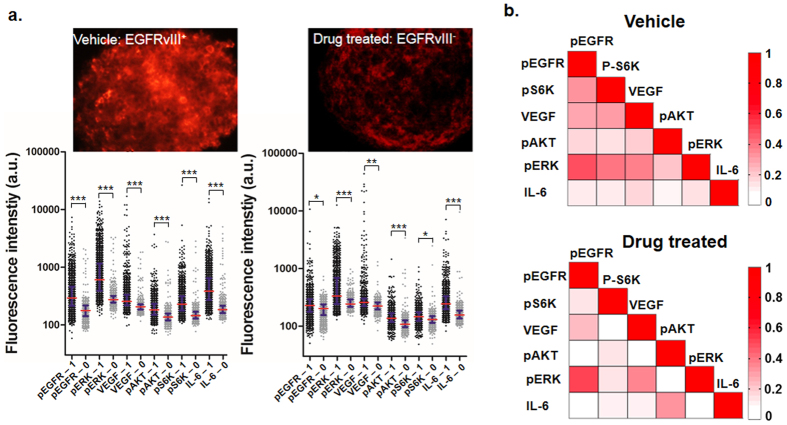
(**a**) Application of the stand-sit microchip to single-cell assays with brain tumor cell line. Fluorescence images of vehicle U87 cells expressing EGFRvIII and the cells after Erlotinib treatment for 2 weeks in neural sphere culture are shown in the upper panel, and their corresponding single-cell protein detection results are shown in the dot plots below. 1 cell and 0 cell results are compared for each protein. *P* values: *P* < 0.05 (*), *P* < 0.01 (**) and *P* < 0.001 (***), with *P* *<* 0.05 considered significant. (**b**) Correlation map of 6 proteins calculated from single-cell data on vehicle cells and drug treated cells. The colormap shown on the right indicates the correlation coefficient. Experiments are repeated two times, but the data in this figure are from the same chip to lower experimental variations.

We also demonstrate the utility of our technology in detecting proteins in single brain tumor cells that express epidermal growth factor receptor variant III (EGFRvIII) and those after drug treatment. We show the ability of assaying drug treated cells with the clamp/SSC assembly without much common laboratory facilities to simulate pre-clinical setting. Cell samples in neurosphere culture were digested into single cells and loaded into the SSC, the procedure of which should be also compatible with that for primary tumor samples. After 4 h on-chip culture, cells were lysed, and both secreted proteins and intracellular proteins were detected by sandwich ELISA on the SSC. The whole process can be performed with the microchip assembly, a pipettor and a few aliquots of molecular biology reagents. The SSC after analysis can be stored for days and transported for scanning and data acquisition. Compared with the vehicle cells, the drug treated cells have much lower expression of all the signaling proteins that promote tumor growth (Fig. 5a). However, blocking tyrosine phosphorylation of EGFR does not completely inhibit PI3K signaling; as shown in Fig. 5b, PI3K pathway signaling and IL-6 signaling are still active in a small portion of cells. The correlation map in Fig. 5b indicates that after drug inhibition, weak correlations of a few protein pairs are lost, while IL-6 and p-Akt are more correlated.

## Discussion

We present a high-throughout, multiplexed single-cell technology based on a microchip/clamp assembly that is portable for single-cell analysis of cancer samples, which is developed towards point-of-care diagnosis at the single-cell level. With the current concept, a tumor sample is digested into single cells and is analyzed by the SSC immediately, while the glass slide can be stored for days and be transported to a core facility for data reading. The feasibility has been testified as we demonstrated that the fluorescence intensity on the glass slide only decreased <10% over a week if it is preserved in a dry environment. Although the method is not quite “sample-to-answer” detection yet, the current technology overcomes certain limitations of other similar technologies. For example, the SSC permits complicated on-chip manipulation such as adding certain substances at a designated time to the on-chip cultured cells, while the whole chip operation is kept simple, and most of signaling protein types at the single-cell level can be quantitated. Another similar portable microchip developed in Shi’s group powerfully allows for codetection of metabolic activity, intracellular functional proteins and genetic mutations from single circulating tumor cells^18^. But on-chip operation may lack quantitative, fine control on this platform. Other valve-less, simple single-cell platforms introduced by Fan’s group and Love’s group are focused on detection of secreted proteins instead of intracellular proteins^43^,^44^,^45^. All these technologies possess distinctive sensitivity which is mainly determined by the cell chamber size and the antibody array quality. In our microchip system, the microchambers are isolated during incubation and after lysis, and the diffusion time is over 2 hours at the stand-open state and should be much longer at the stand-close state. Given the low diffusion rate of proteins, the leakage of proteins to the outside of microchambers would be negligible. But the volume of microchambers at 0.75 nl and antibody density on the array may be the leading factors that influence sensitivity. During the experiments, we observed that cell lysis always increases background noise significantly, while detection of cytokines without cell lysis resulted in much lower background signal. This may be caused by non-specific interaction of numerous cellular contents with the antibody array. To extract the more accurate biological information, we set a threshold for each protein at the mean plus 2 times standard deviation using 0-cell data. This processing can help identify certain positive cell subpopulations. Alternatively, without setting thresholds, other algorithms such as principal component analysis and PLS-DA can intrinsically deal with noisy data to find useful information.

Although intracellular cytokine staining (ICS) and phosphor-flow cytometry (PFC) can be used to study the signaling events, they have certain intrinsic weaknesses. Staining of intracellular cytokine needs blocking of the surface transporters first, which may significantly interfere the activities of intracellular kinase. In our method, cytokine proteins are secreted to the outside of cells, which is more relevant to the real biological process. PFC is normally not quantitative and relies on gating by forward scatter (FSC) and side scatter (SSC) to lower noise, and additional gating by cells without staining is used to select the fluorescent cells. In our case, although we set mean +2 s.d. as the threshold to lower measurement noise, this is by no means the ideal process for low expression cytokines or phosphoproteins. For example, phosphoproteins are normally hundreds to a few thousand copies per cell in low aggressive cells, which is close to the detection limit to our technology and the similar ones using barcode microchips. Setting a threshold may erroneously alter the heterogeneity information.

When the SSC is used to analyze breast CSCs, it shows that the CSCs are more active in uPA secretion, ERK pathway and VEGF production (Fig. 4a). The secretion of uPA is linked to maintaining basal levels of activated ERK signaling in MDA-MB-231^46^; therefore the observed higher levels of uPA with concominant increase in p-ERK1 signal indicate that the ERK pathway was more active for the ALDH^high^ subpopulation. VEGF is an angiogenic factor that induces formation of blood vasculature^31^, a process that promotes tumor growth, and thus increased secretion of VEGF by ALDH^high^ cells may indicate that tumor aggressiveness is related to higher ALDH activity. The clustering result in Fig. 4c also supports that conclusion by showing that oncogenic signaling levels are generally higher in CSCs. Although a cell line is analyzed here, the intercellular heterogeneity is still fairly extensive.

We demonstrate the SSC is also applicable for studying brain tumor cells and profiling the oncogenic signaling of both drug untreated and treated cells at the single-cell level. Although we have not used the real tumor, the neurosphere culture shares certain similarity and preserve molecular characteristics of a tumor. EGFRvIII cells are used in our assay. EGFRvIII is a frequently occurring mutation in primary glioblastoma and renders EGFR constitutively phosphorylated and activated, resulting in fast growth and proliferation of tumor cells^47^. In the U87 EGFRvIII cell line, the majority of cells have the potential to express EGFRvIII. The chemotherapy drug Erlotinib targets the intracellular domain of EGFRvIII and EGFR, and thus the intervention by Erlotinib would presumably block EGFR related pathways such as PI3K pathway and inhibit tumor aggressiveness^48^. However, Erlotinib treatment often leads to drug resistance and recurrence of cancer^49^. In our assay, we found that the coordination between the assayed proteins involved in the PI3K pathway is lowered after drug treatment, and the percentage of cells expressing those proteins is also much lowered. However, the blocking of PI3K activity does not kill the cells in short term and long term either, presumably the drug treated cells may have developed resistance mechanisms by amplifying expression of other growth factor receptors and kinase proteins to counteract the inhibition^3^,^50^. Fig. 5 clearly indicates that the drug treated cells still possess active PI3K pathway signaling, but the correlation between the kinases is significantly changed. Thus, there could be other interconnecting pathways exploited by cells that activate those phosphoproteins to maintain cell growth and proliferation.

In summary, the SSC technology permits full set of complicated on-chip single-cell manipulation and detection of multiple cancer-related signaling proteins in single cells with simple operation, and can be used in a resource-limited setting. We develop this technology towards single-cell point-of-care diagnosis. In addition, the PDMS slab and the glass slide are separable after assay, cells can be potentially further analyzed to obtain genetic information. This portable multiplexed single-cell tool may find broad applications in cancer diagnosis in the near future, as heterogeneity severely hinders the effectiveness of cancer therapies and no clinical single-cell tool hitherto exists.

## Methods

### Microchip Fabrication

The general procedures for fabricating the polydimethylsiloxane (PDMS; Ellsworth Adhesives) microchip by lithography were described previously^24^. Briefly, a multilayer mold was constructed by patterning SU-8 (Microchem) and SPR220 (Dow Chemical Company) photoresists on a 4″ silicon wafer. The first layer is a broad slice of SU-8 2100 (thickness of 100 μm) containing circular holes with the same diameter as the posts. After development, the second layer made of SPR220 (thickness of 3 μm) was fabricated. The second layer formed the mold for the ducts connecting microchannels to microchambers. The third layer, corresponding to the mold for microchambers, was fabricated from SU-8 2015 (thickness of 25 μm). The final SU-8 2025 layer (thickness of 50 microns) formed the mold for the microchannels in a grid design with inverted “bowl” features at the intersections of the gridlines. The bowls have hollow centers that are aligned with the circular holes of the first layer, so that the PDMS posts carved out of the mold attain a height of 150 μm. The features of the 2^nd^, 3^rd^, and 4^th^ layer were allowed to slightly overlap to ensure that the ducts were connected to microchambers and microchannels.

The features of the mold were transferred to PDMS with 10:1 ratio of base to curing agent. The PDMS replica was finally mated with an antibody array to become a functional microchip. The resulting microchip had a fixed height of 75 mm, containing ~6000 microchambers 1000 μm × 30 μm × 25 μm for single cell isolation. A clamp constructed in-house was used to apply force to the chip by turning the adjustable screw (Fig. S1). Varying the application of force using the clamp system allowed for changing the configuration of collapsible PDMS posts between “stand” and “sit” states as well as ducts between “sit-open” and “sit-closed” states.

### Fabrication and Validation of Antibody Array

The antibody barcode array on a glass substrate was prepared with a modified method from our previous publication for patterning the array initially as a DNA array, followed by conversion to an antibody array by hybridization of DNA-antibody conjugates^24^. The PDMS mold for patterning the array was fabricated using standard soft lithography techniques. The mold was attached to a poly-L-lysine slide (Thermo Scientific) by baking at 80 °C, after which a solution consisting of 150 μM amine-modified DNA (Table S1), 1 mM bis (sulfosuccinimidyl) suberate (BS3; Thermo Scientific) and 20% dimethyl sulfoxide (DMSO; Sigma Aldrich) in phosphate buffered saline (pH 7.4) was patterned on the glass slide. Six different oligonucleotide sequences (D, E, F, G, H and I; see sequences in Table S2) were used to generate DNA barcode array. The DNA barcode array was validated (Fig. S2) by hybridization of complementary DNA sequences (D’, E’, F’, G’, H’ and I’) tagged with Cy3. The barcode pattern was visualized by scanning the slide using a GenePix scanner (Molecular Devices).

Capture antibodies for uPA, p-ERK1, VEGF, p-S6K, IL-8, p-EGFR, IL-6, and p-Akt were purchased from R&D Systems (Table S2). The capture antibodies were conjugated to oligonucleotides using a method described previously^51^. Conjugation was performed in two steps using succinimidyl-4-formylbenzoate (S-4FB) and succinimidyl-6-hydrazinonicotinamide (S-HyNic) linkers purchased from Solulink. In the first step, amine groups of 5′-amine modified oligos (D’, E’, F’, G’, H’, and I’) were converted to 4-formylbenzamide groups by reacting with S-4FB. Similarly, capture antibodies were functionalized with aromatic hydrazine groups by reacting primary amine groups on the antibodies with S-HyNic. The formation of the Schiff base by reaction of 4-FB and HyNic at pH 6.0 coupled the antibodies to oligos, thereby producing the conjugates listed in Table S4.

The DNA array was converted to an antibody array by incubating the conjugates at 2.5 μg/ml on the slide at 37 °C. Conversion was always done immediately preceding on-chip protein detection assays. For the cancer stem cell study with MDA-MB-231, the antibody array was patterned using capture antibodies for uPA, p-ERK1, VEGF, p-S6K and IL-8. For the drug treatment study with U87 cells, the array consisted of capture antibodies for IL-6, p-EGFR, VEGF, p-Akt, p-ERK, and p-S6K. The arrays were validated and calibrated by detecting recombinant proteins at concentrations of 10 to 10^4^ pg/mL.

### Cell Culture and Sample Preparation

MDA-MB-231 cells were grown under 5% CO_2_ in Dulbecco’s Modified Eagle Medium (DMEM, Sigma Aldrich) with 10% fetal bovine serum (FBS, Gibco) and 1% penicillin/streptomycin. Cells were passaged every 3 days. MDA-MB-231 cells were stained with Aldefluor following a protocol we optimized for on-chip imaging. Trypsinized cells (9 × 10^5^) were incubated in Aldefluor assay buffer with 4.6 μg/mL activated Aldefluor reagent (Stem Cell Technologies) for 30 minutes at 37 °C. The staining process involves the entry of activated Aldefluor reagent, BODIPY-aminoacetaldehyde (BAAA) into cells, where intracellular ALDH would convert BAAA into BODIPY-aminoacetate (BAA), a charged molecule that is retained in cells when efflux pump activity is inhibited by components of the Aldefluor assay buffer. After incubation, cells were washed with 5 volumes of ice-cold PBS and resuspended in ice-cold Aldefluor assay buffer (3 × 10^6^ cells/mL). Temperature was kept low to minimize the loss of fluorescence intensity due to efflux of BAA, which was favored at higher temperatures. Aldefluor-stained MDA-MB-231 cells exhibited green fluorescence with varying intensities.

The brain cancer cell line U87 was purchased from American Tissue Culture Collection (ATCC). U87 EGFRvIII cells were constructed as reported^52^,^53^. Cells were maintained in neurosphere culturing conditions consisting of DMEM/F12 (Gibco) supplemented with B27 (Life Technologies), Glutamax (Life Technologies), 5 μg/ml heparin (Sigma-Aldrich), 20 ng/ml EGF (Life Technologies) and 20 ng/ml FGF (Life Technologies). Medium was refreshed every 3–6 days. Cells were treated with 5 μM Erlotinib for two weeks to obtain the drug treated cells. Dead cells were removed by centrifuging at 200 rcf in a falcon tube. For immunofluorescence staining, cells were fixed and permeabilized by methanol first, followed by detection by anti-phospho-EGFR antibody (R&D Systems) and labeling by anti-mouse IgG conjugated with Alexa 647 (Life Technologies).

### On-Chip Operation and Protein Detection

The chip was sterilized by washing with 70% ethanol then drying in the sterile environment of a laminar flow hood. The face of the chip containing engraved features was coated with 100 μg/mL collagen (Corning) in deionized water. The collagen-coated chip was mated to a DNA barcode slide, with barcodes aligning with the microchambers. While the chip was in the “stand” state, a blocking solution of 3% bovine serum albumin (BSA) in phosphate buffered saline (PBS) at pH 7.4 was flowed through. During the blocking step, the complete microchip system was assembled by placing the chip in the mechanical clamp, and attaching 200-μL pipette tips to the inlets and outlets. Pipette tips along the inlets served as reservoirs for buffer/medium being supplied to the chip, while those at the outlets collected wash-through. At this point, the chip was in the stand state and bulk flow of the contents was controlled by pipetting and by gravity-induced flow. After blocking, 5 μg/mL of antibody-oligonucleotide conjugates were added to the chip then incubated for 1 h. Hybridization of the conjugates immobilized antibodies onto specific barcodes on the slide, generating the antibody array. Excessive conjugates were washed off with 3% BSA in PBS.

For MDA-MB-231 cells, the cell suspension after Aldefluor staining (Stem Cell Technologies) was immediately loaded into the chip at the stand state. Converting the chip to the sit-closed (SC) state isolated single cells into microchambers, although the random nature of loading also yielded chambers with 0 or >1 cells. After imaging all cell chambers to count the cell number in each chamber, culture medium (10% FBS in DMEM) with 2 μM calcein-AM dye was supplied to the chip in the SC state, then the adjustable screw of the clamp system was rotated at a fixed angle (Fig. S1b) to open ducts (SO state) and to push the channels’ contents into cell chambers. This rapid exchange resulted in washing off the Aldefluor assay buffer from the cell chambers, and calcein-AM (Life Technologies) stained cells were observed in microchambers.

Cells were incubated on-chip for a total of 4 h at 37 °C. Within this duration, secreted proteins were captured by the antibody barcode array. At t = 4 h, images were taken again to observe viable cells (stained with calcein-AM) after incubation. Non-viable cells were excluded from the proteomic analysis. After incubation, cells were lysed by the addition of 6x lysis buffer (Cell Signaling) to the chip, while still at the SO state. A high concentration of lysis buffer was used to account for the dilution of the buffer when mixed with the contents of the cell chambers. The chip was incubated for an additional 2 h to allow the intracellular proteins from cell lysates to be captured by the antibody barcode array.

The microchambers were labeled by adding Cyanine 3 (Cy3) *N*-hydroxysuccinimide (NHS) ester (Lumiprobe) to the microchannels while the ducts were closed off. The NHS ester moiety reacted with amine groups on the poly-L-lysine barcode slide. This step introduced a grid pattern on the barcode slide. The grid pattern assigned spatial addresses to the microchambers, thus specific identities were assigned to every microchamber simply by noting their location on the grid. After this step, the antibody barcode slide was detached from the microchip and washed.

To complete the sandwich immunoassay, biotinylated secondary antibodies (R&D Systems) were incubated on the slide for 1 h. Subsequent incubation with Streptavidin Alexa 647 (SA-647, Life Technologies) and Cy3-tagged DNA (F’-Cy3) labeled the antibody arrays and the reference barcodes, respectively. Upon completing the assay, the slide was gently washed with PBST (0.05% Tween 20 in PBS, pH 7.4; Cell Signaling) and water before scanning.

### Microscopy Imaging and Data Acquisition

Images of the chip and cells after staining were taken systematically on an inverted fluorescence microscope (Olympus IX73) equipped with three fluorescent filter sets: a green fluorescein filter set (U-FF, Olympus; excitation filter 450–490 nm, dichroic 500 nm long pass, emission 520 nm long pass), a yellow Cy3 filter set (U-FF, Olympus; excitation filter 528–553 nm dichroic 565 nm long pass, emission 590–650 nm), and a red Cy5 filter set (U-FF, Olympus; excitation filter 590–650 nm, dichroic 660 nm long pass, emission 665–740 nm). A microscope objective (UPlanApo, Olympus, 4X/0.16; UPlanFLN, Olympus, 10X/0.30/Ph1; UCPlanFLN, Olympus, 20X/0.70/Ph2) was used to collect fluorescence light. A digital camera (Zyla sCMOS, Andor) mounted on the microscope was used to capture images using Andor SOLIS software. Images recorded the number of cells in each microchamber as well as cell total fluorescence which was proportional to ALDH activity. Images were processed with ImageJ (NIH), and integrated density of the gray value was taken as a measure of total cell fluorescence. Mean gray value was measured to quantify the changes in concentrations of Dylight 488 and SA-647 used in diffusion experiments.

Array slides were scanned using Axon GenePix 4400A microarray scanner (Molecular Devices) to quantitate the fluorescent signals. Fluorescence readouts obtained using a Cyanine 5 (Cy5) excitation wavelength (635 nm) correspond to detected proteins, while the fluorescence at the Cy3 (532 nm) wavelength is assigned as the reference. The scanner settings included optical (photomultiplier tube, PMT) gain of 600 and 100% power for Cy5, and PMT gain 450 with 10% power for Cy3.

### On-Chip Cell Viability Testing

MDA-MB-231 cells were loaded into the “stand”-“sit” chip following the steps of the integrated ALDH/functional proteomic assay, but without cell lysis. Upon loading, images of cells were taken, after which the Aldefluor assay buffer was exchanged with culture medium containing the live-cell staining dye, calcein AM (Invitrogen). Cells were counted at t = 0 and t = 4 h of incubation. Microchambers containing greater than or equal to 4 cells were excluded for cell counting, as cells were too crowded to allow optimum nutrition intake.

### Data and Statistical Analyses

Single-cell data were digitized by GenePix Pro (Molecular Devices) and processed by Prism (GraphPad) and Matlab (Mathworks) for comparison of 0 cell data vs. 1 cell data as well as ALDH^high^ and ALDH^low^ subpopulations. Unpaired, two-tailed t-test was used to determine statistically significant differences. A *P* value less than 0.05 is considered statistically significant and is denoted with *, while ** and *** represent *P* < 0.01 and *P* < 0.001, respectively. Error bars on scatter dot plots represent the interquartile range of the sample, whereas red bars denote the median intensity.

Hierarchical clustering was applied to single-cell data above the threshold set for background fluorescence (mean +2 *standard deviation of 0-cell fluorescence). Cluster 3.0 (Stanford University) with TreeView 3.0 (Java) were used to generate heat maps. Data was normalized by median-centering then hierarchical clustering was performed using a Euclidean distance similarity metric with centroid linkage clustering algorithm.

Partial Least Squares (PLS) Toolbox (Eigenvector) in Matlab was used to perform partial least squares discriminant analysis (PLSDA) to relate multiplex protein data of single cells with ALDH activity. PLSDA is a dimension reduction regression in which the orthogonal components are constructed to maximize the sample covariance between the response values (X) and the linear combination of the predictors (Y) or protein expression values. The components, or latent variables, are ranked in a new space with percentiles to describe the behavior of the predictor. PLSDA is selected because of its advantage in handling collinear and noisy large-scale data. It has been widely applied in high-dimensional data processing for clinical outcome prediction, cancer classification, and omics data analysis^38^,^54^,^55^,^56^,^57^. Protein assay signals in our study were designated as “predictors” and were loaded as input X data, while cell total Aldefluor fluorescence was loaded as Y data for prediction. The data set was preprocessed to assign a uniform standard deviation (s = 1) to all variables to correct for the different scales of protein signals versus Aldefluor fluorescence intensities. The weight of each protein in two dominant latent variables was calculated and plotted in 3D by Matlab.

## Additional Information

**How to cite this article**: Ramirez, L. *et al*. Stand-Sit Microchip for High-Throughput, Multiplexed Analysis of Single Cancer Cells. *Sci. Rep.* **6**, 32505; doi: 10.1038/srep32505 (2016).

## Supplementary Material

Supplementary Information
