# Supplementary material for: Stand-Sit Microchip for High-Throughput, Multiplexed Analysis of Single Cancer Cells
Source: Sci Rep. 2016 Sep 1;6:32505. doi: 10.1038/srep32505 (PMC5007481; doi:10.1038/srep32505)
Supplement: Supplementary Information [file srep32505-s1.pdf]

Supporting Information

Stand-Sit Microchip for High-Throughput, Multiplexed Analysis of Single Cancer Cells

Lisa Ramirez[1], Jason I. Herschkowitz[2,3] and Jun Wang[1,2#]

[1]Multiplex Biotechnology Laboratory, Department of Chemistry, University at Albany, State University of New York, Albany, NY 12222

[2]Cancer Research Center, University at Albany, State University of New York, Rensselaer, NY 12144

[3]Department of Biomedical Sciences, University at Albany, State University of New York, Albany, NY 12222

[#] To whom correspondence may be addressed. Email: jwang34@albany.edu

Supporting Figures

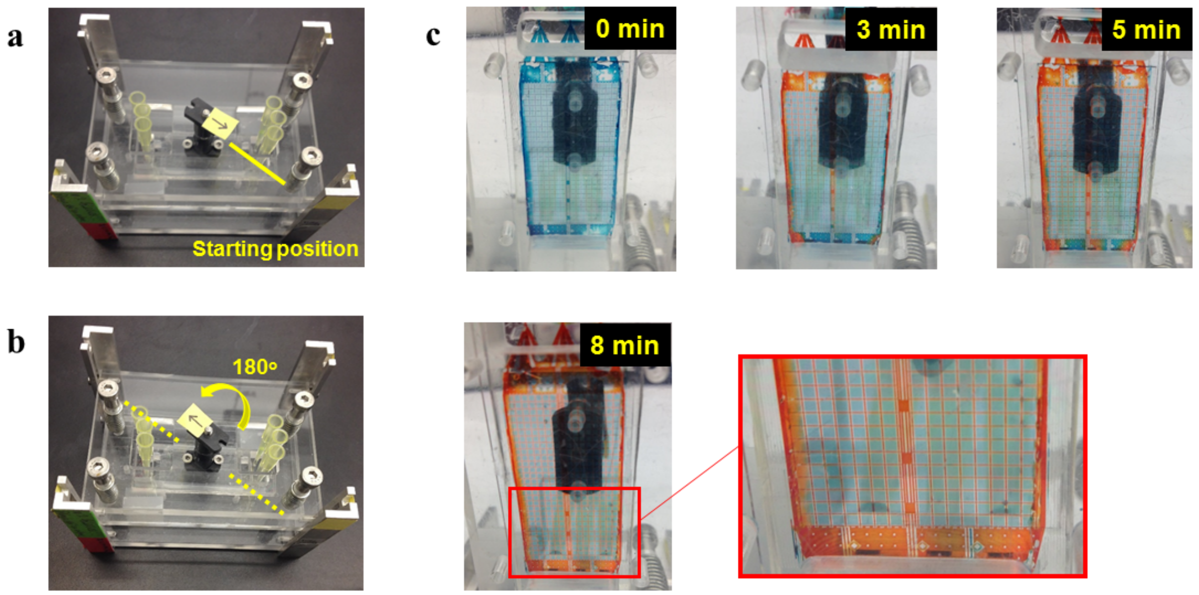

Figure S1. Use of a mechanical clamp system for controlling material exchange within the stand-sit chip. (a) Starting position of the adjustable screw which corresponds to the SC state of the chip. Ducts are closed or fully obscured at this configuration. (b) Rotation of the screw by 180 degrees counter-clockwise yields the SO state. (c) Gravity-induced material exchange confined within channels of the microchip while the clamp system is tilted. Orange food dye traversed the channels of the chip, but without any leakage into the microchambers containing blue dye. In the span of 8 minutes, the blue dye in channels was completely replaced by orange dye.

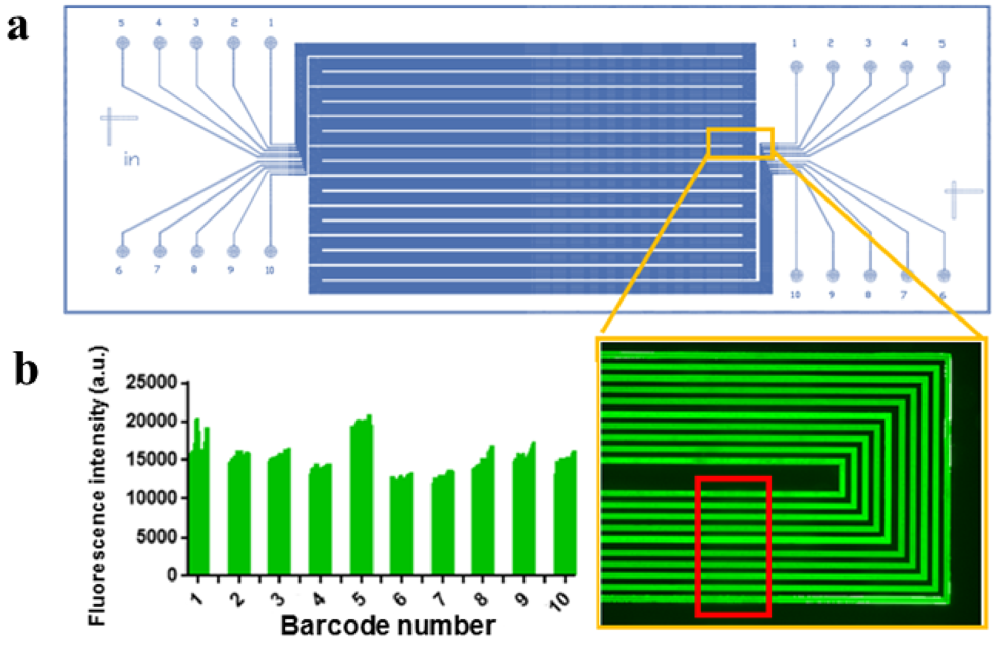

Figure S2. DNA barcode design and validation. (a) AutoCAD design for the barcode mold yielding 10 different barcodes, each stripe with width of 50 µm. (b) Validation of 10 barcodes by hybridization with Cy3-tagged complementary DNA sequences. Fluorescence intensity was measured for the selected rectangular area of the barcode pattern.

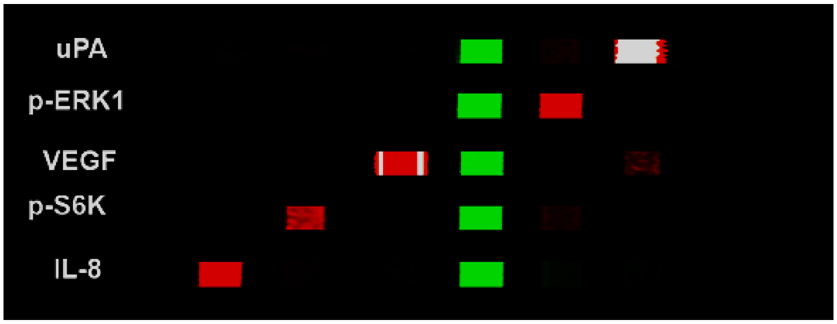

Figure S3. Cross-reactivity of assayed proteins. 6-element barcode arrays were used for sandwich ELISA using a mixture of capture antibody-DNA conjugates and corresponding detection antibodies for all conditions, while using one standard recombinant protein per row (shown in red Cy5 signals) at 5 ng/mL concentration. The green-colored array elements serve as reference signals for the spatial address of the barcodes and were developed by hybridization of Cy3-labeled DNA. The major Cy5 signals for protein detection are confined to the designated barcodes with minor crosstalk. Differences in fluorescence intensities of the Cy5 signals may be attributed to variations in the performance of the recombinant proteins and/or antibody pairs.

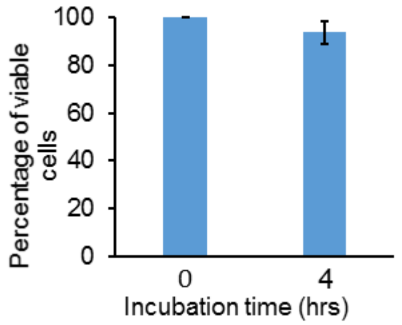

Figure S4. Assessment of on-chip cell viability for the integrated Aldefluor/functional proteomic assay. A column of microchambers (240 chambers with around 50 cells) was chosen randomly and imaged right after loading Aldefluor-stained cells (t = 0 h) in Aldefluor assay buffer. The assay buffer was exchanged with cell culture medium, then cells were incubated for 4 h. The medium was supplemented with 2 µM calcein-AM to facilitate imaging of viable cells. At t=4 h, viability was still >90%, and this suggests that majority of cells retained viability within the incubation period required for co-detection of ALDH and signaling proteins.

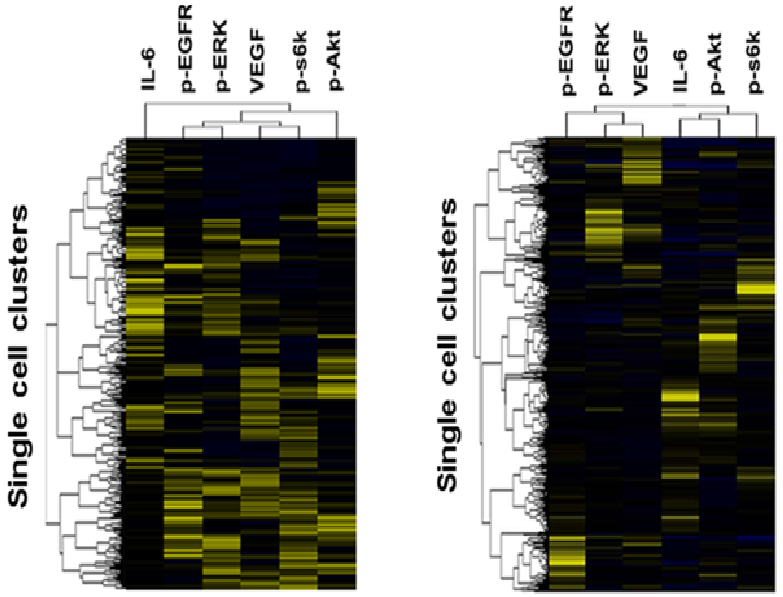

Figure S5. Heatmap of single-cell assays from brain tumor cell lines. Both single cells and proteins are clustered by Euclidean distance. The left and right heatmaps correspond to vehicle EGFRvIII positive cells and EGFRvIII negative cells, respectively.

Table S1. Material Exchange Processes Related to Stand-Sit States of the Chip

State of the Chip	State of the Clamp	Relative Force (F) Applied to the Chip	Height of Ducts	Material Exchange
**Sit-closed**	Screw at starting position	F = 1, constant force	0 µm	Convective flow in microchannels, no flow in microchambers (ducts are closed)
**Transition from sit-closed to sit-open**	Screw rotated 180 degrees from starting position	Start from 1, end at 0<F<1 (force is not constant)	Start from 0, end at 3 µm	Convective flow between microchannels and microchambers (ducts are open)
**Sit-open**	Screw remains rotated 180 degrees from starting position	0<F<1, constant force	3 µm	Diffusive flow between microchannels and microchambers (ducts are open)
**Stand**	No clamp, or screw in clamp is -1800 degrees from starting position	F = 0	No ducts	Convective flow throughout 100 uL bulk volume

Table S2. Sequences of ssDNA oligomers

Name	Sequence
D	5’-AAA AAA AAA AAA AAT GGT CGA GAT GTC AGA GTA-3’
D’	5’-AAA AAA AAA AAA ATA CTC TGA CAT CTC GAC CAT-3’
E	5’-AAA AAA AAA AAA AAT GTG AAG TGG CAG TAT CTA-3’
E’	5’-AAA AAA AAA AAA ATA GAT ACT GCC ACT TCA CAT-3’
F	5’-AAA AAA AAA AAA AAT CAG GTA AGG TTC ACG GTA-3’
F’	5’-AAA AAA AAA AAA ATA CCG TGA ACC TTA CCT GAT-3’
G	5’-AAA AAA AAA AAA AGA GTA GCC TTC CCG AGC ATT’3’
G’	5’-AAA AAA AAA AAA AAA TGC TCG GGA AGG CTA CTC-3’
H	5’-AAA AAA AAA AAA AAT TGA CCA AAC TGC GGT GCG-3’
H’	5’-AAA AAA AAA AAA ACG CAC CGC AGT TTG GTC AAT-3’
I	5’-AAA AAA AAA AAA ATG CCC TAT TGT TGC GTC GGA-3’
I’	5’-AAA AAA AAA AAA ATC CGA CGC AAC AAT AGG GCA-3’

Table S3. Antibodies used for the protein assay

Antibody	Source	Oligo Sequences
Anti-human uPA/urokinase	R&D Systems, DY1310	D'
Anti-human/mouse/rat phospho-ERK1 (T202/Y204)	R&D Systems, DYC1825	E'
Anti-human VEGF	R&D Systems, DY293B	G'
Anti-human/mouse/rat phospho-p70 S6 kinase (T421/S424)	R&D Systems, DYC8965	H'
Anti-human CXCL8/IL-8	R&D Systems, DY208	I'
Anti-human phospho-EGFR (ErbB1)	R&D Systems, DYC1095B	D'
Anti-human IL-6	R&D Systems, DY206	F'
Anti-human/mouse phospho-Akt1 (S473)	R&D Systems, DY2289C	I'

Supporting Methods

Characterization of On-Chip Mass Transport Processes (Sit State)

The chip underwent several "sit' states (sit-closed or SC, sit-open or SO) for the dual assay and we sought to characterize the mass transport processes associated with each state as well as the transition from sit-closed (SC) to sit-open (SO) states.

1. Sit-closed

   We visualized the convective mass transfer process for the flow of solutions from the inlets to the outlets of the chip using blue and orange dyes. We first flooded the chip with a blue dye solution at the stand state, then set the chip to SC at t=0, after which a second dye solution was fed to inlets and the chip was oriented vertically. Images were taken every two minutes in the span of 10 minutes (Fig. S2).

2. Transition from sit-closed to sit-open

   This transition is associated with opening the ducts and bridging the channels and chambers. We first filled the chambers with PBS, followed by the channels with 1 ug/mL Dylight 488 at SC. The adjustment screw was then rotated by 180 degrees to reach SO, then images of the chip were taken in a span of 15 minutes.

3. Sit-open

a. Diffusion of small molecules (~1 kDa) from channels to microchambers, then from microchambers to channels

   Dylight 488 was used as a model small molecule, with a molecular weight of ~1 kDa. The chip was first filled with PBS at the stand state, then set to SC, followed by SO at t = 0. Dylight solution (1µg/mL) was allowed to flow through the channels in 10 minutes to exchange PBS from the channels. Afterwards, the chip was oriented horizontally to allow simple diffusion. Images were taken every 20 minutes.

   Diffusion from microchambers to channels was visualized using the same method, except that chambers were first filled with Dylight, and channels with PBS.

b. Diffusion of small molecules (~1kDa) from microchambers to channels

   Streptavidin conjugated with Alexa 647 fluorophore (average molecular weight of 60 kD) was used to visualize diffusion of large molecules into microchannels while the chip was in the SO state. The chip was filled with 40 µg/mL SA-647 in PBS while in the stand state, after which the chip was converted to SC then SO where PBS was used to wash away the SA-647 from the microchambers. After washing for 10 minutes, images were taken at 1-hour intervals to visualize the diffusion of SA-647 from microchambers into microchannels.

The diffusion of secreted and intracellular proteins in the microchambers was calculated using Einstein–Smoluchowski equation for diffusion:

$$L = \sqrt{2Dt} \quad (1)$$

Where *L* is the mean displacement of the molecule within time interval *t*, and *D* is the diffusion coefficient (in water). The diffusion time was calculated for distances 100 µm up to 500 µm, which is the distance from the center of a microchamber to the duct openings. For the diffusion of Dylight 488 (~1 kDa), we used D = 3 x 10^6 cm^2/s based on the diffusion coefficient of Cy5 (~0.7 kDa) in water a 25 °C, which was estimated to be 3.6 x 10^6 cm/s. Thus, the diffusion throughout the 1 mm microchamber only needs ~0.5 h, and 3 min through ducts. However, the small cross-section ducts significantly limit the amount of molecules exchanging between microchannels and cell chambers, as the diffused amount can be roughly estimated by movement rate x cross section area. The typical proteins (10-50 KDa) in our assay need 1-2 hours to diffuse from one end to the other end. They might have been captured by the antibody array once released by the cells. Thus, after cell lysis, we set 2 h for proteins sufficiently depleted by the antibody array.
